# The Role of Epigenetics in the Progression of Clear Cell Renal Cell Carcinoma and the Basis for Future Epigenetic Treatments

**DOI:** 10.3390/cancers13092071

**Published:** 2021-04-25

**Authors:** Javier C. Angulo, Claudia Manini, Jose I. López, Angel Pueyo, Begoña Colás, Santiago Ropero

**Affiliations:** 1 Clinical Department, Faculty of Medical Sciences, European University of Madrid, 28005 Madrid, Spain; 2 Department of Urology, University Hospital of Getafe, Getafe, 28907 Madrid, Spain; 3 Department of Pathology, San Giovanni Bosco Hospital, 10154 Turin, Italy; claudia.manini@aslcittaditorino.it; 4 Department of Pathology, Cruces University Hospital, 48903 Barakaldo, Spain; joseignacio.lopez@osakidetza.eus; 5 BioCruces-Bizkaia Health Research Institute, 48903 Barakaldo, Spain; 6 Foundation for Biomedical Research, Innovation of University Hospitals Infanta Leonor and South-East, 28003 Madrid, Spain; angel.pueyo@salud.madrid.org; 7 Heath Science PhD Program, UCAM Universidad Católica San Antonio de Murcia, Guadalupe de Maciascoque, 30107 Murcia, Spain; 8 Biochemistry and Molecular Biology Unit, Department of Systems Biology, University of Alcalá, 28805 Alcalá de Henares, Spain; begona.colas@uah.es (B.C.); santiago.ropero@uah.es (S.R.)

**Keywords:** renal cell carcinoma, biomarker, DNA methylation, epigenetics

## Abstract

**Simple Summary:**

The accumulated evidence on the role of epigenetic markers of prognosis in clear cell renal cell carcinoma (ccRCC) is reviewed, as well as state of the art on epigenetic treatments for this malignancy. Several epigenetic markers are likely candidates for clinical use, but still have not passed the test of prospective validation. Development of epigenetic therapies, either alone or in combination with tyrosine-kinase inhibitors of immune-checkpoint inhibitors, are still in their infancy.

**Abstract:**

Clear cell renal cell carcinoma (ccRCC) is curable when diagnosed at an early stage, but when disease is non-confined it is the urologic cancer with worst prognosis. Antiangiogenic treatment and immune checkpoint inhibition therapy constitute a very promising combined therapy for advanced and metastatic disease. Many exploratory studies have identified epigenetic markers based on DNA methylation, histone modification, and ncRNA expression that epigenetically regulate gene expression in ccRCC. Additionally, epigenetic modifiers genes have been proposed as promising biomarkers for ccRCC. We review and discuss the current understanding of how epigenetic changes determine the main molecular pathways of ccRCC initiation and progression, and also its clinical implications. Despite the extensive research performed, candidate epigenetic biomarkers are not used in clinical practice for several reasons. However, the accumulated body of evidence of developing epigenetically-based biomarkers will likely allow the identification of ccRCC at a higher risk of progression. That will facilitate the establishment of firmer therapeutic decisions in a changing landscape and also monitor active surveillance in the aging population. What is more, a better knowledge of the activities of chromatin modifiers may serve to develop new therapeutic opportunities. Interesting clinical trials on epigenetic treatments for ccRCC associated with well established antiangiogenic treatments and immune checkpoint inhibitors are revisited.

## 1. Current Management of Renal Cell Carcinoma

Renal cell carcinoma (RCC) is the seventh most common form of human neoplasm, with an incidence of 10 new cases per 100,000 inhabitants in Western Europe and United States. Its incidence is steadily rising due to increased incidental detection. Among the genitourinary tumors RCC is the one with highest mortality, with approximately 76% global 5-year survival rate, and accounts for 2% of global cancer deaths in the world [1]. Probably the main clinico-pathological parameters, that predict prognosis in this malignancy, are nuclear grade, tumor stage, cell type, tumor architecture, and tumor diameter [2]. However, Fuhrman grade, node involvement, number of different metastatic sites, and whether cancer-directed surgery is recommended and performed are the major factors involved in the prediction of prognosis in metastatic RCC [3].

Histological variants characterize different subtypes within RCC [4]. The most common is clear cell RCC (ccRCC) that accounts for a total 75% of cases. Papillary RCC (pRCC) is the second in terms of frequency, approximately 20%. Chromophobe RCC and its benign counterpart oncocytoma account each for approximately 5%. Other rarer tumors enter in the differential diagnosis of solid renal masses [5]. Both ccRCC and pRCC arise from the proximal tubule while chromophobe RCC (chRCC) has an origin in the distal part of the nephron. Each type has different morphology but also different genetics and behavior. Tumor grade has prognostic value for ccRCC. An individual tumor can have mixed histology and different subtypes can occasionally appear within the same kidney. Heterogeneity of RCC stands at the molecular, genomic, histopathological, and clinical levels [6,7]. It explains how appropriate tumor sampling is needed for a correct identification, and implies great difficulty for the development of accurate diagnostic and prognostic markers. In fact, among the many candidates investigated, no marker of ccRCC has reached the clinic today.

Sensitive and specific molecular markers for the diagnosis and monitoring of RCC are lacking [8,9]. Tumor heterogeneity of the disease, worsened by specific histological subtypes, also affect the search for accurate biomarkers [10]. Likely earlier detection and better clinical monitoring of this malignancy might help to improve its prognosis [11]. Compared to other subtypes, ccRCC has a more unfavorable prognosis. Although, it is curable when diagnosed early, no screening strategy is being used. Small renal masses are often detected by imaging studies performed for other reasons and tend to be treated by nephron-sparing surgery, although ablation or active surveillance when diagnosed in an elder population is increasingly used. In this clinical situation, imaging monitoring to evaluate clinical progression is mandatory in the absence of reliable molecular tumor marker of disease progression. Many candidates, including a number of epigenetic markers such as DNA methylation profiling, have been proposed both for screening and prognostic evaluation [12,13,14,15,16,17,18,19]. In fact, DNA methylation presents itself as a potentially strong biomarker to predict aggressive behavior and risk of tumor recurrence in patients with apparently less aggressive renal tumors [20].

Radical nephrectomy or partial nephrectomy, that imply total or partial removal of the kidney, are the main therapeutic basis of local and locally advanced disease [21]. Approximately 30% of the patients develop metastases, either synchronically or during follow-up, and for the last decades have been treated with adjuvant or palliative classical immunotherapy with interferon-α2b (IFN-α2b), high-dose interleukin-2 (IL-2), systemic targeted therapies including tyrosine kinase inhibitors (TKI) targeting the VEGF signaling axis (sorafenib, sunitinib, pazopanib, axitinib, lenvatinib, and cabozantinib) or mTOR inhibitors (everolimus and temsirolimus) or the anti-VEGF monoclonal antibody bevacizumab. First-line options for metastatic ccRCC included sunitinib, pazopanib or the combination bevacizumab plus interferon-α and second-line options were axitinib and cabozantinib. Despite all treatment efforts, advanced disease implies very low survival rates. Median duration of response was 9 months for the first-line setting and 6 months for the second-line. In the absence of toxicity most of these agents have been given sequentially until further disease progression. Cytoreductive nephrectomy was also advocated whenever possible in cases with metastatic onset to reduce the tumor burden and avoid further metastatic seed.

Many studies are currently evaluating the combination of anti-VEGF therapy with the new generation of immunotherapy agents T-cell immune checkpoint inhibitors (ICI), that include antibodies against programmed cell death protein ligand-1 (PDL1) avelumab and atezolizumab, antibodies against programmed cell death protein 1 (PD1) nivolumab and pembrolizumab, and the inhibitor of cytotoxic T-lymphocyte-associated protein 4 (CTLA-4) ipilimumab. Blockade of the PD1–PDL1 axis promotes T cell activation and immune killing of cancer cells. ICIs have very recently become first-line standards of care as improved survival for ipilimumab and nivolumab combined has been demonstrated in the intermediate and poor-risk group, while pembrolizumab plus axitinib combination is recommended, for both unfavorable and favorable disease. Cabozantinib remains a valid alternative for the intermediate and high-risk group. To summarize, in patients previously treated with TKIs that progress, nivolumab, cabozantinib, axitinib, or the combination of ipilimumab and nivolumab appear indicated; while in patients already treated with ICI, any VEGF-targeted therapy previously unused together with ICI therapy appears a valid option [22,23,24,25].

PDL1 immunohistochemical expression in tumor cells or in tumor-infiltrating mononuclear cells (TIMC) has been thoroughly evaluated as biomarkers for the prediction of ICI response in metastatic disease. However, PDL1 expression is not a good predictive marker and does not serve to assign the most convenient therapy. Response rates are better in PDL1 positive tumors, but PDL1 negative ones also respond [26]. It is important to note that the role of CTLA-4 expression in TIMC has been forgotten to evaluate response to ICI. Many issues are responsible for the failure to develop predictive biomarkers, to name dynamic expression, and the aforementioned heterogeneity within primary tumor, and between primary and metastases [27]. Seric levels of PDL1 could be a novel prognostic factor in ccRCC and also a predictor of response to TKI-based therapy [28]. It is a paradox that despite the fact that treatments for metastatic ccRCC are targeted, the approach for immunotherapy is far from being targeted.

Abnormal epigenetic patterns will give new opportunities to develop novel therapies in RCC. Some drugs targeting the epigenetic system are currently under investigation; however, strategies that combine therapies targeting epigenetic machinery with conventional therapies for this malignancy, either targeting tyrosine kinases, mTOR or immune checkpoints at different combinations are still at infancy. Maybe closer is the practical utility of epigenetic therapies to solve or delay therapy resistance in ccRCC, and also to identify the populations in which prolonged response to a certain therapy could be expected. Hopefully the introduction of biomarkers into clinical practice will allow personalized patient care for renal cancer [29,30].

## 2. Epigenetics of Clear Cell Renal Cell Carcinoma

Epigenetics studies the inheritable phenotype resulting from changes in gene expression without alteration of the DNA sequence. As such, cancer epigenetics deals with the inheritable but reversible changes associated with gene expression dysregulation that manifest in a pre-malignant phenotype with the genomic sequence unaltered. Interest in epigenetic alterations associated with ccRCC provides an optimal scenario in the search for new tumor markers in this malignancy, and also to develop new treatment strategies facilitated by the reversibility of epigenetic modifications. The main epigenetic mechanisms are DNA methylation, chromatin remodeling, post-translational histone modifications, short-noncoding RNAs, also known as microRNAs (miRNA), and long-noncoding RNAs (lncRNA) [31,32,33].

Interestingly, all the epigenetic modifications work together to regulate chromatin structure and gene expression. Disruption of the epigenetic homeostasis may derive from deregulation of epigenetic modifiers. That means altered epigenetic modifications can be explained by changes in expression and function of epigenetics writers, erasers, and readers. These changes can be due to genetic alterations, linking genetics, and epigenetics in carcinogenesis.

Translational epigenetic research is a growing field to identify and validate new markers leading to personalized medicine. Among the many epigenetic changes and signatures identified in RCC, aberrant promoter methylation of more than 200 genes have been reported and more than 120 miRNAs are deregulated [34]. According to several recent systematic reviews of diagnostic DNA methylation biomarkers in this disease, none of the biomarkers proposed exceeds level of evidence III, which means their clinical utility is limited [34,35]. Promising biomarkers should be validated not only in sample banks, but also in prospective clinical trials before their use can be generalized [29]. In every case after the publication of a potential biomarker, prospective cohort studies that increase the evidence are lacking. Additionally, more standardized methodology is needed to facilitate reproducibility, and that hinders clinical translation [35]. Bias in sample selection and handling, DNA methylation detection methods and genomic location of the assay can also bring confounding results. In addition, the selection of normal tissue for comparison with neoplasia can be problematic because aberrant promoter methylation is an early event in carcinogenesis allowing its detection in normal appearing tissue surrounding the tumor. Finally, inter-individual study comparison is most often lacking for further biomarker validation.

However, there is no doubt that DNA methylation and histone modification patterns have a crucial role in the regulation of global and local gene expression and may play an outstanding role both in carcinogenesis and tumor progression. Firstly, epigenetic deregulation can lead precursor cells to proliferate and block their differentiation as seems to occur in germ cell malignancies [36]. This is of primary importance in childhood renal kidney tumors like nephroblastoma [37]. Probably the most interesting epigenetic mechanism in ccRCC stands in common mutations in chromatin regulator genes that complement the inactivation of Von Hippel Lindau (*VHL*) tumor suppressor gene (TSG), and Hypoxia-inducible factors (HIF) pathway that allow tumor cell survival in a characteristic status of pseudo-hypoxia. *VHL* gene is frequently inactivated in sporadic ccRCC by mutation, loss of heterozygosity, or promoter hypermethylation [38]. In addition, several miRNAs have been associated with VHL-HIF pathway. In particular, downregulation of MIR-30c has been associated with loss of VHL in RCC [39]. However, little is known about the relationship between lncRNAs and VHL–HIF pathway. A study comparing lncRNA expression profile in VHL-wild type and VHL-mutant RCC cell lines and demonstrated that LncRNA-SARCC is differentially regulated in a VHL dependent manner in RCC cell lines and tumor samples [40].

Recent genome-wide sequencing studies have revealed a number of mutations of genes coding for epigenome modifiers and chromatin remodelers, like *PBRM1* (40%), *SETD2* (10%), *KDM5C* (10%), *KDM6A* (1%), and *BAP1* (10–15%). Most of the mutations of histone modifier genes described in ccRCC are truncating and inactivating mutations producing loss of functions [41,42]. Apart from *VHL* mutations these are among the most common somatic genetic abnormalities encountered in renal tumors [43,44]. Very interestingly, 90% of sporadic ccRCC are affected by a 50Mb deletion on chromosome 3p where not only *VHL* but also *PBRM1*, *BAP1,* and *SETD2* are located and act as a functional gene group [43]. The function of these epigenetic modifiers stands in DNA repair and maintenance of genomic integrity by regulating splicing and other processes like cytoskeletal regulation that also contribute to genomic stability. *KDM5D* and *KDM6C* located on the Y chromosome, are homologs of the X-lined genes *KDM5C* and *KDM6A*, and are often deleted in male patients with ccRCC [43]. Understanding how chromatin modifiers contribute to RCC tumorigenicity will serve to develop the basis for therapeutic interventions as well. Finally, it is important to recognize that epigenetic modifications work together and can also regulate one another, thus diversifying their function. This regulatory network has been defined as epigenetic crosstalk.

Epigenetic changes can be evaluated in samples obtained with minimal invasion (e.g., urine or plasma), and this represents an added attraction to introduce epigenetic studies in the clinic. Obtaining DNA non-invasively from renal cells in urine is an ideal scenario for epigenetically based detection of ccRCC. Additionally, DNA can be obtained from fresh tumor or paraffin-embedded tissue. Liquid biopsy from direct washing of fresh biopsies can be an optimal method as well, to evaluate epigenetic changes that would facilitate accurate detection, tumor subtype determination, and evaluation of prognosis as well [45]. More recently detection of RCC using plasma and urine cell-free DNA methylomes has also been confirmed [46,47].

The potential of renal cancer epigenomics has been investigated later than in other urologic malignancies, but the understanding of how genomics and epigenomics disturb biologic functions and determine intratumor heterogeneity will help to explain the complex reality of RCC and the differences in molecular cancer phenotypes [48]. The growing field of knowledge to determine the real impact of altered epigenetic patterns and their role in the diagnosis, monitoring, classification, prognosis, and treatment of kidney cancer is the main objective of this review.

### 2.1. Abnormal DNA Methylation

DNA methylation is the most widely studied epigenetic modification so far, and consists of the addition of a methyl group to the Cytosine within the CpG dinucleotide. This epigenetic modification is a reversible process regulated by writers, DNA methyl transferases (DNMT), erasers, and Ten-eleven translocation (TET). The maintenance of DNA methylation through replication is ensured by DNA (cytosine-5)-methyltransferase 1 (DNMT1) but de novo DNA methylation is mediated by DNMT3A and DNMT3B [49]. DNMTs transfer the methyl group from S-adenosyl methionine (SAM) to carbon-5 of the cytosine. The proportion of CpG dinucleotides in the human genome is lower than expected from the abundance of cytosine and guanine. The distribution of the CpGs is not uniform and concentrates in short areas, called CpG islands, located mainly in the promoter regions of approximately 60% of known genes [50]. Promoter DNA methylation is a mark of transcriptional repression, while gene body DNA methylation is generally associated with a permissive transcriptional state. This epigenetic modification is crucial in several physiologic functions, including X-chromosome inactivation, silencing of tissue specific genes, imprinting and genomic stability, and changes due to senescence. In normal cells, around 80% of CpGs are methylated, including repetitive genomic sequences and transposons but most of the CpG islands are unmethylated allowing gene expression when necessary, but this methylation pattern is altered in malignant transformation. Two major changes occur in cancer affecting DNA methylation: global DNA hypomethylation of the genome and aberrant hypermethylation of the promoter region of TSGs. Age and environmental changes also have a strong effect on DNA methylation. The methylation of a gene promoter causes gene silencing through a transcription failure.

DNA hypomethylation primarily affects repetitive sequences and pericentromeric regions that are methylated in normal cells. Loss of methylation at these elements in cancer may result in chromosomal instability and mutations [50,51]. In addition, the hypomethylation of CpG sites has been associated with the over-expression of oncogenes within cancer cells and with deregulation of proteins involved in the complex balance between methylation and the maintenance of the chromatin structure [50]. Hypermethylation of CpG islands located in the promoter regions of some TSGs prevents gene expression and, therefore, its protective role in the development of tumors. Gene silencing by promoter hypermethylation in cancer has been studied in depth and affects important functions for cell cycle, DNA repair, cell adhesion and invasion, apoptosis, miRNA expression, metabolism of carcinogens, and response to hormones. In particular, silencing of negative regulators of cell cycle (*RASSF1* and *KILLIN*), activation of Wnt pathway by suppression of Wnt antagonists (*SFRP1*, *SFRP2*, *SFRP5,* and *WIF-1*), TGF-β activation by promoter methylation of negative regulators (*GATA-3*, *GREM-1,* and *SMAD-6*) and silencing pro-apoptotic genes (*APAF-1*) are the most important mechanisms that explain why gene hypermethylation plays an important role in development and progression of RCC [33,52].

Characterizing methylation patterns and signatures in cancer is one of the bases for the desired personalized medicine in the search for biomarkers. First of all, unlike mutations and other genetic alterations, methylation always occurs in defined regions of DNA and can be precisely detected with resolution [53]. Secondly, every tumor type has a specific methylation profile, referred to as hypermethylome, somehow different from that of other neoplasia. Thirdly, methylation-specific PCR (MSP) derived methods enable a fast, simple method to detect methylated alleles of a certain gene in samples with low tumor content and even in biological fluids [54,55]. However, among the limitations to generalize application of epigenetic markers in RCC is also cell type specificity and the aforementioned heterogeneity of this malignancy [56].

#### 2.1.1. DNA Methylation as Marker of RCC Diagnoses

Aberrant DNA methylation is an early event in carcinogenesis, thus DNA methylation biomarkers has been implemented for the diagnosis of a wide range of malignancies including prostate, colorectal, and pulmonary neoplasia [57]. Regarding RCC, LINE1 methylation levels in leukocyte DNA measured prior to cancer diagnosis has been identified as a biomarker of RCC risk among male smokers [58]. Diagnostic DNA methylation biomarkers, despite being very promising for RCC, have not reached clinical practice yet [35]. However, it is well known that some genes including *APC*, *BNC1*, *CDH1*, *ECAD*, *GSTP1*, *KTN19*, *IGFBP1*, *IGFBP3*, *MGMT*, *PTGS2*, *p14ARF*, *p16/CDKN2a*, *p16INK4a*, *RASSF1A*, *RARB2*, *SRFP*, *TIMP3*, *UCHL1,* and *VHL* are silenced in RCC by DNA methylation and this could be useful for the diagnosis of RCC in tumor tissue, serum, or urine samples, both in the familiar and sporadic forms [12,59,60,61,62,63,64,65,66]. Concordance between serum and tissue DNA hypermethylation profile has been proved, especially with grade and tumor stage [67].

#### 2.1.2. DNA Methylation as Marker of RCC Subtyping

Classification of sporadic RCC into different histologic subtypes is allowed by multigene quantitative methylation profiling because DNA methylation signatures reveal cell ontogeny and establish differences between precursor cells in the nephron [18,68]. *VHL* methylation is restricted to ccRCC. *RASSF1A* and *SPINT2* are more frequently methylated in pRCC [63,69,70] while *COL1A1* and *IGFBP1* hypermethylation is more common in ccRCC [62,63]. *CDH1* methylation is significantly higher in ccRCC than in chRCC or oncocytoma [63], important discrimination due to the benign nature of oncocytoma. In fact, data from The Cancer Genome Atlas (TCGA) revealed that of all RCC subtypes, oncocytoma and chRCC are the most similar but, what is even more interesting, a signature of 30 hypermethylated genes distinguishes oncocytoma from chRCC [48,71] involved, among others, in Wnt, MAPK, and TGFβ signaling [48]. From a practical perspective the distinction between oncocytoma and ccRCC can be performed with very high sensitivity and specificity using a three-gene promoter methylation panel (*OXR1*, *MST1R,* and *HOXA9*) and this distinction could be very useful to allow unnecessary overtreatment if performed in preoperative biopsies before nephrectomy [14].

#### 2.1.3. DNA Methylation as Marker of RCC Prognosis

Although classical histologic parameters are the most valuable tools to evaluate prognosis, nuclear grade and staging have some limitations to precisely predict the clinical outcome in RCC. DNA methylation-based classification is highly relevant for clinical management of RCC as it serves to identify the prognosis of different epigenetic subtypes. In fact, DNA methylation data can classify inherent tumor heterogeneity into specific-prognosis subgroups according to DNA methylation at promoter sites identified in The Cancer Genome Atlas (TCGA) network [72]. Integrated genomic and epigenomic analysis revealed significant correlations between the total number of genetic aberrations and total number of hypermethylated CpGs [73]. In recent years, several groups have used multi-omic data analysis to reveal groups of differentially methylated and expressed genes in surgically resected specimens of RCC or in the open data of ccRCC in TCGA (TCGA Research Network). The evidence generated confirms cluster analysis based on genome-wide promoter methylation serves to identify panels of methylated genes associated to ccRCC disease progression [17,34,72,73,74,75,76,77,78,79,80,81,82,83]. Some of these panels have been validated in an independent retrospective cohort and some have been incorporated into prognostic risk score models to enhance their prognostic biomarker effect [77,78]. However, none has been prospectively validated in multicenter studies [35]. Additionally, a methylated site signature useful for prediction of prognosis has been identified for pRCC, validated in the TCGA and GEO cohorts and incorporated in a nomogram that predicts an individual’s risk of survival in pRCC [80]. Again, this panel has not yet been revalidated prospectively.

Some of the panels focus mainly on two or more genes for prognostic classification of ccRCC patients [17,74,81,82,83]. Other investigations evaluate tumor prognosis and progression based on analyzing the functional role of a particular gene and the likely mechanisms involved. In this sense, promoter CpG methylation of γ-catenin is considered an independent predictor for survival and disease progression [84]. Other hypermethylated genes associated with worse RCC disease-specific survival are: GATA Binding Protein 5 (*GATA5*), that codify for a DNA-binding transcription factor [85,86]; Gremlin 1 (*GREM1*), related to cytokine activity and bone morphogenic protein [87]; HIC ZBTB Transcriptional Repressor 1 (*HIC1*), related both to DNA-binding transcription factor activity and histone deacetylase binding [88]; Junction Plakoglobin (*JUP*), related to protein homodimerization activity and protein kinase binding [84]; neural EGFL like 1 (*NELL1*), linked to calcium ion binding [76]; Protocadherin 8 (*PCDH8*), also related to calcium ion binding [89]; Phosphatase and Tensin Homolog (*PTEN*), related to protein kinase binding [90]; Ras Association Domain Family Member 1 (*RASSF1A*) that encodes a protein similar to the RAS effector proteins [91,92]; sarcosine dehydrogenase (*SARDH*), associated to oxidoreductase activity [93]; and Secreted Frizzled Related Protein 1 (*SFRP1*), related to G protein-coupled receptor activity [94,95]. Very recently some methylated genes with prognostic value in pRCC have also been described [96].

### 2.2. Methyl-Binding Proteins

Methyl-binding proteins (MBP) are readers of DNA methylation. They bind to methylated CpG nucleotides and induce gene silencing by recruiting repressor complex containing histones deacetylates (HDAC) linking the DNA methylation with histone modifications [97]. The MBP family is composed of human proteins MBD1, MBD2, MBD3, MBD4, and MECP2. Each of them, with the exception of MBD3, is capable of binding specifically to methylated DNA. Among them MBD2 is the MBP with highest affinity for methylated DNA. MBD2 alters the structure of chromatin and mimics chromatin remodeling or modification factors, and may serve as transcriptional repressor or activator, depending on the cell context [98]. MBD2 upregulation has been reported in many different malignancies such as RCC and is associated to neoplastic progression, with potential as a biomarker and a therapeutic target [99].

### 2.3. Post-Translational Histone Modifications

**Figure 1:**
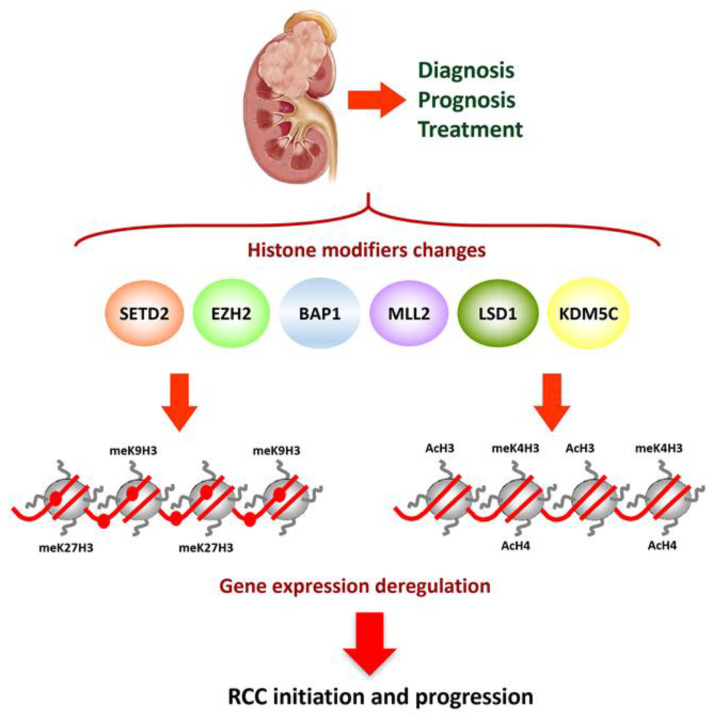
Summary of the altered histone modifiers genes in RCC. Histone modifiers changes induce gene expression deregulation and thus RCC initiation and progression. These alterations can be used as biomarkers for RCC diagnosis and prognosis. H3Ac, global acetylation of histone H3; meK9H3, methylated lys9 of Histone H3; meK27H3, methylated lys27 of Histone H3; meK4H3, methylated lys4 of Histone H3.

Chromatin is a complex nucleoprotein structure formed by DNA, histones, and other proteins. The DNA is wrapped around an octamer of histones (2H2A, 2H2B, 2H3, and 2H4) that is the repeating unit of chromatin. The chemical modifications of amino acids in the external tail of histone molecules determines changes in the chromatin structure. Lysine residues can undergo methylation, acetylation, or ubiquitylation, while arginine residues can be methylated and the serine residues phosphorylated [100]. The best studied histone modifications are acetylation and methylation of lysine present at the N-terminal tails of histones H3 and H4. These histone modifications are reversible and result from the balance of two enzymatic activities: histone acetyltransferases (HAT) and histone deacetylases (HDAC) regulate histone acetylation, while histone methyltransferases (HMT) and histone demethylases (HDMT) regulate histone methylation. The combination of all histone modifications builds the histone code that regulates all chromatin functions [39,101,102] (Figure 1).

Post-translational histone modifications play a very important role in regulating, not only chromatin structure but also gene expression. Changes in the acetylation and methylation state of histone tails convert loosely packed regions with high transcriptional activity into densely packed ones with scarce activity. Acetylation is associated with a more open conformation and is related with active transcription. The effect of methylation depends on the residue affected and also on the degree of methylation; the methylation of H3K4, H3K36, and H3K79 activates transcription while methylation of H3K9, H3K27, and H4K20 produces repression [33,103].

Global histone modifications are likely markers of cancer prognosis in RCC [104]. Diminished H3K4me2 and H3K18Ac levels worsen prognosis [105] while acetylated histone H3 (H3Ac) immunostaining inversely correlates with staging, Fuhrman grade, and tumor progression [106]. Similarly, it has been suggested that H3K9Ac and H3K18Ac levels could monitor patients with RCC after surgery, but as far as we know these likely markers have not been confirmed in prospective validations [107,108]. H3K27 methylation levels also correlate with established clinical-pathological variables and survival in RCC [104]. Additionally, H3K27me1/-me2/-me3 staining is significantly more intense in pRCC than in ccRCC, and H3K27me3 levels are higher in oncocytoma than in RCC [104]. The mono-methylation of histone H3 on lysine 27 (H3K27me1) plays key roles in the cellular processes, interacts with the DNA sequence of the miRNAs and regulates the transcription of miRNAs [109]. The enrichment analysis of molecular function shows H3K27me1-associated miRNAs are linked to RNA binding and protein binding involved in the transcription and translation regulation. As a result, the biological roles of the H3K27me1 appear closely related to miRNAs downstream [109].

Histone modifications alterations in cancer can be explained by changes in the activity or expression of histone modifiers and readers, and these changes could be valuable in cancer management. Different studies indicate that changes in histone modifications in RCC are related to hypoxia and the prognostic relevance of associated alterations. There is a strong relation between hypoxia and epigenetic regulation, especially histone modifications. One of the mechanisms involved in the epigenetic-altered landscape in RCC related to hypoxic effect is the regulation of Jumonji domain containing histone demethylases by the mediator of hypoxic response HIFα [110]. A number of genes that encode histone-modifying enzymes are mutated in ccRCC [41,111]. Inactivating mutations described for *SETD2* (H3K36 methyltransferase), *KDM5C* (H3K4 demethylase), *KMD6A* (H3K27 demethylase), *MLL2* (H3K4 methyltransferase), Polybromo 1 (*PBRM1*), BRCA1 Associated Protein-1 (*BAP1*) remain among the most interesting epigenetic mechanisms for ccRCC progression. This merits a brief description of the function of some of them.

*SETD2*, located at chromosome 3p near *VHL*, *BAP1,* and *PBRM1* genes, is inactivated in approximately 10% of RCCs which results in global reduction in the histone mark trimethylation of lysine 3 of histone H3 (H3K36me3) and a global loss of DNA methylation across the genome. This gene is involved in genome stability as trimethylation of H3K36 by *SETD2* is required for DNA repairing system through both homologous recombination repair and mismatch repair [112,113]. DNMT3B-mediated de novo DNA methylation occurs at the intron of genes marked with H3K36me3 but not those lacking H3K36me3.

Mutations in the switching defective/sucrose nonfermenting (SWI/SNF) chromatin remodeling complex gene *PBRM1* are identified in approximately 40% of ccRCC [114]. The SWI/SNF complex mobilizes nucleosome and modulates chromatin structure, thus affecting transcription, DNA repair, cell proliferation, and cell death. It is essentially a key regulator of gene expression and is associated with numerous transcription factors [115]. Inactivation of *PBRM1* causes enhanced cell proliferation and cell migration. It also regulates the expression of genes the products of which are involved in cell adhesion, like E-cadherin [116]. Thus, inactivation of the *PBRM1* TSG amplifies the HIF-response of *VHL* negative ccRCC [117,118]. *PBMR1* has been proposed as a tumor suppressor gene in ccRCC since its re-expression in ccRCC cell lines lacking *PBMR1* function decreased cell proliferation by upregulating genes involved in cell adhesion and apoptosis [116]. *PBRM1* is implicated in the regulation of gene expression through its bromodomains. In particular, *PBRM1* contains six bromodomains that bind acetylated histones, thus serving as a reader for H3K14Ac, and target SWI/SNF chromatin remodeler complex to DNA regulatory regions [119]. In addition, PBRM1 also binds to acetylated p53 and facilitates its binding to regulatory elements at the promoter genes regulated by p53 in ccRCC [120].

*BAP1* is also located very close to *SETD2* and *PBRM1* genes and is mutated in more than 10% of ccRCCs. BAP1 forms a multiprotein complex with breast cancer type 1 (BRCA1) susceptibility protein to regulate DNA damage response and cell cycle control, but its exact function in ccRCC remains largely unknown [43].

Lysine Demethylase 6A (*KDM6A*) and Lysin Demethylase 5C (*KDM5C*) are X-linked histone demethylase-coding genes located near each other in Xp11. *KDM6A* codifies a protein that demethylases lysine 27 in histone 3 (H3K27) and is mutated in only 1% of ccRCCs, while *KDM5C* encodes H3K4 demethylase and its mutation is present in approximately 10% of ccRCCs [41,121]. In urothelial bladder cancer KDM6A-deficient cells depend on EZH2, a HMT that methylates lysine 27 on histone H3 (H3K27). Inhibition of EZH2 has been suggested as an effective therapeutic approach to *KDM6A*-mutated tumors [122]. *KDM5C* acts as TSG and its deficiency results in genomic instability and aggressive forms of ccRCC [123]. Interestingly both *KMD6A* and *KDM5C* are considered escape from X-inactivation tumor suppressor or EXIT genes. Their homologues on chromosome Y, *KDM6C,* and *KDM5D* are downregulated due to loss of chromosome Y in 40% of male patients with ccRCC [124]. This fact is most likely involved in male predominance of ccRCC.

Lysine-specific histone Demethylase 1A (LSD1 or KDM1A) can demethylate both lysine 4 and lysine 9 of histone H3 (H3K4me and H3K9me), thereby acting as a co-activator or a co-repressor, depending on the context. It has been found as a part of several histone deacetylase complexes, and silences genes by functioning as a histone demethylase. Conversely, it can also act as coactivator of androgen receptor (AR) dependent transcription and is regulated by AR activity in renal cells [125]. The mammalian homolog of LSD1, LSD2 has been associated with tumor stage and metastasis in ccRCC and, thus proposed as a biomarker for ccRCC progression. Moreover, LSD1 and LSD2 expression was correlated in metastatic ccRCC [126].

Enhancer of zeste homolog 2 (*EZH2*), as has been previously mentioned, codify for a HMT acting as a transcriptional repressor through regulating the methylation of histone H3 at lysine 27. Not much evidence exists regarding EZH2 in ccRCC but high tumor and initial reports suggested EZH2 level was associated with less aggressive tumor phenotypes and favorable prognosis [127]. However, more recent evidence has confirmed high EZH2 expression correlates with poor overall survival in RCC, especially in advanced disease by promoting VEGF expression and cell proliferation while inhibiting apoptosis [128,129]. In agreement with these data, EZH2 represses the expression of E-cadherin through increased levels of H3K27me3, promoting epithelial mesenchymal transition (EMT) and metastases [130].

These studies point out it is interesting to pay attention to the clinical significance of mutations in histone or chromatin modifiers. Mutations in *SETD2* and *KDM5C* are mutually exclusive, as are mutations of *PBRM1* and *BAP1* [43]. *BAP1* or *KDM5C* mutations in ccRCC associate with aggressive disease, high Fuhrman grade, and metastatic at presentation (Figure 1), that imply worse prognosis and instantaneous activation of mTOR signaling [117,131]. However, mTOR activation in *PBRM1* mutated tumors occurs after long latency periods. Additionally, the clinical significance of *SETD2* and *PBRM1* mutations is not well known [43,132,133].

### 2.4. miRNAs

miRNAs are small non-coding RNAs of approximately 22 nucleotides in length implicated in posttranscriptional regulation of gene expression. miRNAs regulate a wide spectrum of cellular processes acting as oncogene or as tumor suppressors of the genes they regulate [134]. A number of functional studies have revealed deregulated miRNA (either upregulation or downregulation) involved in cell cycle regulation, apoptosis, cell adhesion, and extracellular matrix or metabolism with a key role in RCC [111,135,136,137]. In this sense, miR-21 is silenced by promoter methylation in RCC, and its expression inhibits RCC growth through regulating LIVIN, a member of the inhibitor of apoptosis proteins [138].

Numerous reports suggest circulating miRNAs have the potential to be used as biomarkers in patients with RCC. However, findings are diverse, probably due to methodological differences and histological variations in the study cohorts. Initial studies evaluating the implications of serum miRNAs gave conflicting results [139,140]. Currently, the use of two or more miRNAs for diagnosis and molecular classification of RCC is well accepted, supporting miRNA signatures as clinical tools [141]. Most miRNAs are tandemly clustered and co-expression patterns for miR-8, miR-199, miR-506, and other families are downregulated in ccCRC [135].

Different miRNAs are deregulated in RCC. Upregulation of miR-1233 was observed but no prognostic implication could be proved [139]. miR-378 and miR-451 combined serve to identify cancer with 81% sensitivity and 83% specificity [142]. Similarly, miR-210 has 81% sensitivity and 79% specificity for RCC diagnosis [143]. Combining miR-155 upregulation and miR-141 downregulation improves discrimination of ccRCC [144]. However, the best combination reported in terms of diagnostic accuracy could be miR-141 and miR-200b, with 99% sensitivity and 100% specificity [145]. This panel also distinguished chRCC from oncocytoma with 90% sensitivity and 100% specificity [145].

Regarding the prognostic role of miRNAs, overexpression of miR-221 and miR-32 are predictors of RCC mortality [146,147]. Similarly, miR-30a-5p downregulation, probably due to aberrant promoter methylation, is common in ccRCC and can be evaluated both in tumor tissue and urine samples to predict metastatic dissemination and worse survival [148]. Members of the miR-200 family and miR-205 promote EMT and reduced transcription and expression of E-cadherin [149]. They are also induced by bone morphogenetic proteins, part of the TGFβ superfamily of proteins, that antagonizes EMT [150]. miR-454 accelerates RCC progression via suppressing methyl-CpG binding protein 2 (*MECP2*) expression, which may provide a novel potential target of RCC treatment in the future. MiR-454 inhibition and *MECP2* overexpression could both decrease the proliferative, migrative, and invasive abilities of RCC cells and also serve as an independent prognostic factor in RCC [151].

In summary, profiling miRNA in RCC preludes development of new tumor markers [141,151,152,153] but probably even more interesting is the fact that many miRNAs, such as miR-21, miR-155, miR-214, miR-31, and miR-146a, have been implicated in the regulation of immune and stromal cells, and in the modulation of the host immune response [154]. miRNA signatures may be implicated in radio and chemosensitivity and also to predict the response to TKI therapy [141]. Unfortunately, miRNAs occur in a wide spectrum of diseased and benign conditions and are far from being specific for ccRCC, and this limits the possibilities for their use in clinical practice.

### 2.5. lncRNAs

Long non-coding RNAs are a class of transcripts longer than 200 nucleotides that do not codify for proteins and are emerging as regulators of important cellular functions. Although their ultimate function is not very well known, several studies suggest they are involved in apoptosis, cell migration, and cell cycle, and play very critical roles in gene expression regulation, including gene transcription, post-transcriptional regulation, and epigenetic regulation. Differential expression of lncRNAs has been identified in RCC and normal renal tissue [155,156,157] but only a few of these lncRNAs have been studied in depth.

HOX transcript antisense intergenic RNA (HOTAIR) has been proposed as oncogene silencing several TSGs working together with EZH2 and H3K27 histone mark [158]. HOTAIR favors the metastatic process of RCC by upregulation of the histone demethylase KDM6B and its target gene *SNAI1* involved in EMT [159]. More interesting is the lncRNA H19 that is expressed only during embryogenesis, but re-expressed triggered by HIFα in neoplastic renal cells but not in normal kidneys. H19 is implicated, among others functions, in epithelial to mesenchymal transition (EMT) and mesenchymal to epithelial transition (MET) strongly suggesting an oncogenic role in RCC. In addition, H19 is overexpressed in tumor tissues and has been proposed as an independent predictor for the clinical outcome of RCC patients [160].

DNA methylation-deregulated and RNA m6A reader-cooperating (DMDRMR) is another lncRNA recently recognized to facilitate tumor growth and metastasis in ccRCC. DMDRMR binds insulin-like growth factor 2 mRNA-binding protein 3 (IGF2BP3) to stabilize target genes, including the cell cycle kinase CDK4 and several extracellular matrix components (*LAMA5*, *COL6A1,* and *FN1*) [161]. The cooperation between DMDRMR and IGF2BP3 regulates target genes in an m6A-dependent manner and may represent a potential diagnostic, prognostic, and likely therapeutic target in ccRCC.

Another lncRNA important in RCC is KCNQ1 downstream neighbor (KCNQ1DN), downregulated both in neoplastic tissue and cell lines. In vivo experiments with nude mice showed that KCNQ1DN overexpression repressed both the growth of xenograft tumors and the expression of the oncogen *c-Myc*, thus representing a novel target for future therapeutic options in RCC [96]. Reduced expression of KCNQ1DN is also observed in Wilms’ tumor [162].

### 2.6. RNA Methylation

Recent studies also show that RNA methylation serves to epigenetically regulate biological functions. The N6-methyladenosine (m6A) RNA methylation is the most frequent, abundant, and conserved form of RNA methylation reported both in messenger RNAs and lncRNAs. Other well-characterized RNA modifications are 5-methylcytosine (m5C), N7-methylguanosine (m7G), and pseudo-uridine [163,164]. Genome wide changes in gene expression have been reported due to reversible changes in m6A methylation [165]. Same as DNA methylation or histone modifications, m6A methylation is regulated by several methyltransferases, demethylases, and other RNA binding proteins. Methyltransferases involved in the generation of the m6A modification of RNA are m6A writers, while demethylases causing m6A removal are termed m6A eraser. Many RNA binding proteins, including IGF2BP1, IGF2BP2, IGF2BP3, YTHDF1, YTHDF2, YTHDF3, YTHDC1, YTHDC2, HNRNPC, HNRNPA2B1, and RBMX, act as m6A readers, and this regulatory process plays a critical role in stem cell differentiation, development and tumor progression [166,167]. The body of evidence regarding RNA methylation in RCC is still scarce but the expression of some m6A RNA methylation regulatory genes (*IGF2BP3*, *KIAA1429,* and *HNRNPC*) have been recently described as independent predictors of prognosis in pRCC [168]. Other studies point out the expression of RNA methylation modifiers as biomarkers of RCC subtyping. VIRMA and YTHDC2 mRNA expression levels were lower in chRCC and pRCC compared to ccRCC [169].

## 3. Epigenetic-Based Therapeutic Opportunities in ccRCC

Development of epigenetic therapies has been under extensive clinical investigation for the last two decades and may become a promising strategy to restore silenced gene expression both in malignant and non-malignant disease [149,170,171]. The rationale of an epigenetic treatment should consist in reprogramming the pattern of gene expression in cancer cells to result in the induction of apoptosis or in the loss of cell capacity for uncontrolled proliferation and tumor growth, also making cancer more susceptible to conventional therapies [172]. Epigenetic therapy targets three different protein categories: writers, enzymes that establish epigenetic marks; erasers, enzymes that remove epigenetic marks; readers, proteins that recognize epigenetics modifications, and, when recruited to these marks, bring in other protein complexes to exert the desired function.

In the last decades, most of the studies have focused on the use of writers (DNMTs, HATs, and HMTs) and erasers (TET, HDACs, and HDMs) as therapeutics targets, but in recent years a number of studies show the potential use of epigenetic readers as new therapeutic targets. This group of proteins include the bromodomain-containing family of proteins that recognize acetylated lysine residues, the chromodomain-containing proteins that bind to methylated histones, and MBDs, mentioned previously, that bind to methylated DNA [171,173].

Until now DNMT and HDACs inhibitors have been approved by the US FDA for the treatment of hematologic malignancies and myelodysplastic syndromes. These and other drugs with the capacity to inhibit DNMT (decitabine, zacitidine, and guadecitabine) or HDAC (vorinostat, panobinostat, romidepsin, entinostat, belinostat, and AR-42) are being investigated in solid malignancies for their potential to reactivate the expression of silenced TSGs [170,171,174]. There are great expectations for the therapeutic potential and pharmacologic development of these and other agents in early clinical studies in urologic cancer, and more specifically in RCC [149,175]. The role of nutritional interventions affecting epigenetic changes has also been taken into account in breast and prostate cancers [176], but not so far in RCC. The development of new drug alternative for ccRCC has been very promising in the last decades but we can say epigenetic therapy for kidney cancer remains in its infancy.

Future development combination therapies may follow the lead of hematologic malignancies and investigate epigenetic treatments in cases in which current antiangiogenic treatments or immunotherapies (mainly TKIs or ICIs) have failed. However, currently, only phase I/II clinical trials on single-agent or combined therapies for RCC have been completed and the response rate observed is poor and disappointing, with only few patients simply reaching stable disease (Table 1).

### 3.1. DNMT Inhibition Alone or in Combination with Other Therapies

DNMT inhibitors (DNMTi) are cytidine analogues that block the DNMT activity when incorporated into DNA and also induce their degradation. So, DNMTi produce passive DNA demethylation and induce the expression of genes that have been silenced by promoter DNA methylation, thus reactivating silenced TSGs in cancer. The exposure of different tumor cells to low doses of DNMTi cause apoptosis, reduced cell cycle activity, and decreased stem cell function [194].

Azacytidine (Dacogen^®^) and decitabine (Vidaza^®^) are approved by the FDA for the treatment of hematologic malignancies and myelodysplastic syndromes. Guadecitabine (SGI-110), a next-generation hypomethylating agent, is also used in patients with relapsed or refractory acute myeloid leukemia with acceptable efficacy and tolerability profile [195]. Additionally, a phase III trial to evaluate guadecitabine as second-line in patients with myelodysplastic syndromes or chronic myelomonocytic leukemia previously treated with hypomethylating agents is being conducted (EudraCT 2015-005257-12). A rational design of new combination strategies to further exploit the epigenetic mode of action of these two drugs in different areas of clinical oncology was proposed, especially in combination approaches with other anticancer strategies [196].

#### 3.1.1. Azacytidine (5-Azacytidine)

Epigenetic therapy is a promising potential therapy for solid tumors. Integrative expression and methylation data analysis of 63 cancer cell lines (breast, colorectal, and ovarian) after treatment with the DNMTi azacytidine demonstrated significant enrichment for immunomodulatory pathways. These results suggest the possibility of a broad immune stimulatory role for DNA demethylating drugs in solid malignancies [197]. On the other hand, suppressed cell proliferation (>50% reduction in colony formation assay) with azacytidine therapy was detected, both in cell lines with VHL promoter methylation and also in some RCC cell lines without *VHL* TSG methylation, thus suggesting that multiple methylated TSGs might determine the response to demethylating therapies [198].

A phase I trial enrolled 55 patients with advanced neoplastic disease, that included two patients with RCC, to evaluate the combination of azacytidine subcutaneously administered with oral valproic acid. One patient with RCC presented a stable disease for 6 months with a significant increase in histone acetylation. Grade 1 and 2 toxicities were reported [187]. Another phase I trial was performed to evaluate the side effects and best dose of recombinant interferon alfa-2b together with azacytidine for patients with stage III or stage IV melanoma or stage IV kidney cancer that cannot be removed by surgery (NCT00217542). Results have not been published. A phase II trial was specifically intended to evaluate low dose decitabine plus interferon alfa-2b in advanced renal cell carcinoma (NCT00561912) but was terminated early due to slow accrual and unavailable treatment agent. Another study evaluated the effectiveness of azacytidine and bevacizumab in advanced RCC (NCT00934440) with the intention to identify the maximum tolerable dose and assess toxicity. Overall, three different doses were evaluated for each drug. Dose for azacytidine ranged between 35 and 75 mg/m^2^/day for 7 days. All patients presented adverse effects of different degree. Time to progression registered was 5.6 months. Results have not been published.

#### 3.1.2. Decitabine (5-Aza-2′-Deoxycytidine)

Preclinical evidence with the DNMTi decitabine is abundant in renal cancer cell lines. Decitabine inhibits the proliferation of RCC cells via G2/M cell cycle arrest by suppressing p38-NF-κB activity [199]. It also induces apoptosis by regulating the Wnt/β-catenin signal pathway through re-expression of *sFRP2* gene [200]. Additionally, combined treatment with decitabine and valproic acid, a HDAC inhibitor, synergistically inhibits cell growth and migration in ccRCC cell lines [201]. These evidences support targeting DNA methylation with decitabine to treat advanced RCC.

Monotherapy with decitabine was investigated in a phase I study at different doses from 2.5 to 20 mg/m^2^ on days 1–5 in 31 patients with refractory malignancies, including three patients with RCC. Decreased DNA methylation after treatment was evidenced both in tumor and in peripheral blood mononuclear cells. Decitabine also decreased DNMT1 and induced tumor apoptosis [188].

Another phase I trial which evaluated sequential low-dose decitabine plus high-dose IL-2 presented some interesting results in modulating the toxicity and anti-tumor activity of immunotherapy in melanoma, but not in RCC. In this study decitabine caused grade 4 neutropenia lasting more than a week in most patients, and a trend toward a higher incidence of toxicity with increasing decitabine doses was evidenced [189]. The combination of low-dose decitabine with IFNα2b was also evaluated in advanced RCC (NCT00561912), but results have not been reported.

Resistance of RCC to the apoptosis-inducing effects of IFNs was postulated to result from epigenetic silencing of genes by DNA methylation [202]. Decitabine and selective depletion of DNMT1 by phosphorothioate oligonucleotide antisense were used to reverse silencing, in cells resistant to apoptosis induction by IFNα2 and IFNβ. The proapoptotic tumor suppressor *RASSF1A* was reactivated by DNMT1 inhibitors in the cell lines investigated and this was associated with demethylation of its promoter region [203].

The combination of anticancer agents and epigenetic drugs sustains a novel therapeutic strategy. The effectivity rate of chemotherapy for RCC is very low and the high expression of certain drug transporters in the kidney, like the human organic cation transporter OCT2, is partly responsible for this multidrug resistance. Combined treatment using the DNMT inhibitor decitabine and the HDAC inhibitor vorinostat significantly increased the expression of OCT2 in RCC cell lines, which sensitized these cells to oxaliplatin [204]. In this sense, a phase II trial with decitabine combined with oxaliplatin in patients with advanced RCC (NCT04049344) is currently recruiting patients in Zhejiang Cancer Hospital, with the intention of evaluating whether decitabine sensitizes RCC cells to oxaliplatin.

#### 3.1.3. MG98

Another inhibitor of DNMT, the antisense oligodeoxynucleotide MG98 was intravenously administered at a dose of 360 mg/m^2^ twice weekly for three consecutive weeks out of four in 17 patients with advanced RCC receiving a median of two cycles with no objective responses. Mild hematologic toxicity, elevation of transaminases, fatigue, fever, and nausea were observed [190]. Despite the disappointing results, MG98 was investigated in combination with IFNα2b in patients with advanced RCC [191]. Another phase-II trial explored two schedules of MG98 with IFNα2b and described frequent disease stabilization and partial response in one case [205].

### 3.2. HDAC Inhibition Alone or in Combination with Other Therapies

HDAC inhibitors (HDACi) are approved for cutaneous T-cell lymphoma and peripheral T-cell lymphoma. They have dose and compound dependent pleiotropic effects. They induce epigenetic effects either through histone acetylation or by influencing the acetylation status of nonhistone or non-nuclear proteins. A synergy between DNA demethylation and histone deacethylase inhibition has been confirmed to re-express genes silenced in cancer cells [206]. However, from the clinical perspective, some compounds have followed a more productive clinical investigation than others, but today none is approved to treat ccRCC.

#### 3.2.1. Vorinostat

Clinical trials with HDACi in RCC have given mixed results. A phase I trial evaluated the anti-tumor activity of vorinostat (SAHA) as oral agent in 14 patients with advanced RCC (NCT00278395) and showed toxicity in 50% of the cases and 14% serious adverse events. Another study (NCT00324870) evaluated oral vorinostat with becacizumab and observed 18% response rate, mainly partial responses, with an acceptable toxicity and a median overall survival of 13.9 months, thus suggesting clinical activity [177].

A phase I study of sorafenib and vorinostat in patients with advanced solid tumors with expanded cohorts in RCC and non-small cell lung cancer (NSCLC) used oral vorinostat 200–400 mg to establish the recommended phase II dose (NCT00635791). Although tolerable in other tumor types, sorafenib associated to vorinostat was not found tolerable without dose reductions or delays in RCC and NSCLC patients. No complete response was seen but minor responses were observed in RCC [207]. Another dose-limiting toxicity trial with vorinostat plus isotretinoin (NCT00324740) was also performed in 12 patients with recurrent or advanced RCC, of which 33% suffered well tolerated adverse effects, mainly anorexia and weight loss.

Since AKT activation is a possible mechanism of resistance to mTOR inhibitors, adding vorinostat (or another HDACi) was proposed as a route to circumvent AKT-mediated resistance to mTOR inhibitors in experimental studies performed on synovial sarcoma cells [208]. The combination of sirolimus and vorinostat has yielded preliminary anticancer activity in patients with refractory Hodgkin lymphoma, perivascular epithelioid tumor, and hepatocellular carcinoma [178]. Based on these findings another study explored the combination of HDAC and mTOR inhibition in RCC and other solid malignancies. In total, 13 patients with RCC (10 ccRCC and 3 pRCC) were treated with vorinostat and ridaforolimus. Using a dose escalation design, various dose combinations were tested concurrently in separate cohorts. Dosing was limited by thrombocytopenia. Two patients, both with papillary RCC, maintained stable response 54 and 80 weeks, respectively [179]. Additionally, a phase I study with dose finding and extension cohorts using pembrolizumab and vorinostat in patients with advanced or metastatic RCC, urothelial cancer or prostate cancer (NCT02619253) has concluded recruitment, but results are under evaluation.

#### 3.2.2. Panobinostat

Preclinical studies with the pan-deacetylase inhibitor panobinostat (LBH589) have shown induced cell cycle arrest and apoptosis in renal cancer cells and a reduction in tumor size using xenografts mice models [209]. A phase II study was performed to evaluate the activity of panobinostat in refractory renal carcinoma (NCT00550277). In total, 20 patients with advanced ccRCC who had received previous therapy with at least one angiogenesis inhibitor and one mTORi were treated with panobinostat 45 mg orally, twice a week, and evaluated every 2 months. Panobinostat was generally well-tolerated but 30% experienced serious adverse effects. There were no objective responses and all patients progressed or stopped treatment within the first 4 months [180].

A synergistic activity of dual HDAC and mTOR inhibition was confirmed in Hodgkin lymphoma and multiple myeloma cell lines [210,211]. A phase I, dose-finding trial for everolimus combined with panobinostat in advanced ccRCC was performed (NCT01582009). Overall, 21 patients completed this trial which was recently published. Oral everolimus 5 mg daily and panobinostat 10 mg 3 times weekly (weeks 1 and 2) given in 21-day cycles was the maximum tolerated dose. Improved clinical outcomes were not demonstrated as the median time to disease progression was 4.1 months [181].

Synergistic effects have been observed in the combination of TKi, such as imatinib, dasatinib, or sorafenib, with an array of HDACi including vorinostat, romidepsin, or panobinostat [212]. As an example, combination therapy with panobinostat and sorafenib proved to significantly decrease vessel density and tumor volume, and also to increase survival in hepatocellular carcinoma xenografts [213]. Regarding RCC, a phase I study of panobinostat in combination with sorafenib in soft tissue, renal and lung cancers (NCT01005797) was started in 2009 and, with a long history of changes and latest version submitted on 2017, its findings have not yet been reported.

#### 3.2.3. Entinostat (MS-275)

Entinostat reverts retinoid resistance by reverting Retinoic acid receptor β2 (RARβ2) epigenetic silencing in a human RCC model and has a synergistic anti-tumor activity in combination with 13-cis-retinoic acid compared with single agents, suggesting that the combination of HDACi and retinoids represents a novel therapeutic approach for RCC [214]. This observation led to a phase I study with entinostat in combination with 13-cis-retinoic acid in patients with metastatic or advanced solid tumors or lymphomas (NCT00098891). The combination was reasonably well tolerated and the recommended doses were 4 mg/m^2^ once weekly for entinostat and 1 mg/kg/day for 13-cis-retinoic acid. However, no tumor response was evidenced [182].

There are two very interesting trials that are evaluating the combination of entinostat with IL-2. Both are active trials that hopefully will be completed by 2024. One is a phase I/II trial that studies the side effects and best dose of entinostat when given together with IL-2 and the clinical evolution of metastatic RCC with this regime (NCT01038778) [183]. The other is also a phase I/II multicenter, randomized, open label study between high dose IL-2 (3 courses of high dose interleukin 600,000 units/kg administered IV every 8 h on Days 1–5 and Days 15–19, maximum 28 doses) vs. high dose IL-2 (same dose) plus entinostat (5 mg orally given every 2 weeks starting on day 14) in ccRCC (NCT03501381). These trials have been prolonged because the clinical management with high-dose IL-2 has been abandoned with the advent of antiangiogenic and immune-checkpoint inhibiting drugs.

Consequently, two new trials that evaluate entinostat in combination with more actual therapies for ccRCC are currently open. One of these trials, still recruiting patients, evaluates the combination of atezolizumab with entinostat and bevacizumab in patients with advanced RCC (NCT03024437). This study will assess the immunomodulatory activity of entinostat in patients receiving the PD-L1 inhibitor atezolizumab. Additionally, the combination with bevacizumab provides an effective VEGF inhibition to potentiate the immune response and anti-tumor effect induced by atezolizumab [25]. The overall hypothesis is that entinostat will increase the immune response and anti-tumor effect induced by the PD-L1 inhibition by suppressing Treg function, based on the hypothesis that low dose HDACi will have a suppressive function on Tregs but not on effector T-cells. The dose of entinostat starts with 1 mg and is escalated up to 5 mg. The proposed dose and schedule for atezolizumab and bevacizumab follows the standard of the phase III study IMmotion151 (NCT0242082) [215].

The other active clinical trial on the association between HDACi and ICI investigates entinostat with nivolumab plus ipilimumab in previously treated RCC (NCT03552380). This is a phase II, open-label, safety, pharmacodynamic and efficacy study radiologically assessed for patients with metastatic RCC who have progressed on ipilimumab plus nivolumab regimen. The trial starts with a dose finding study for oral entinostat. Following the first 4 cycles of multiple combination treatment ipilimumab will be discontinued, and treatment with entinostat and nivolumab continued until disease progression or prohibitive toxicity. Anti-tumor activity is being assessed every 6 weeks.

#### 3.2.4. Other HDACi

Other compounds with HDACi activity have been investigated and, although selected for preclinical investigation, their pharmacological development has not been completed. Depsipeptide, a cyclic peptide, was isolated from Chrombacterium violaceum during a screening program for anti-oncogene agents. It exerts potent anti-tumor activity against human tumor cell lines and xenografts [216]. A phase II study was performed in patients with metastatic RCC but showed insufficient activity and investigation was abandoned [184].

Romidepsin (FK228) also showed anti-proliferative activity in vitro against multiple mouse and human tumor cell lines and in vivo in human tumor xenograft models [185], but an exploratory phase II trial evaluating its activity and tolerability in patients with metastatic RCC progressive following or during immunotherapy (NCT00106613) was undertaken but results have not been communicated.

Belinostat (PXD101) is another HDACi that has been investigated in patients with advanced refractory solid tumors including mainly colorectal cancer. Stable disease was observed in 39% of the patients included and, among them in 1 of 6 patients with RCC [186]. However, no further investigation has been performed with this compound in RCC.

AR-42 is another HDACi currently investigated in patients with multiple myeloma and T- and B-cell lymphomas [217]. Inhibition of pancreatic cancer cells by regulating p53 expression, inducing cell cycle arrest, particularly at the G2/M stage, and activating multiple apoptosis pathways has been demonstrated [218]. Combined AR-42 and pazopanib have been investigated in advanced sarcoma and RCC (NCT02795819). Of 6 patients recruited, 4 were evaluated for response, and stabilization of disease was confirmed in 2; however, the trial was interrupted because of unacceptable toxicity.

### 3.3. Other Epigenetic Therapies

A more targeted epigenetic therapy based on strategies other than demethylation and histone deacethylase inhibition has been sought after for decades. The strategies investigated include silence miRNAs that are overexpressed, such as, for example anti-mRNA oligonucleotides, miRNA-mask antisense oligonucleotides, and miRNA sponges to restore the expression of miRNAs that are downregulated. Some studies point out the use of miRNAs as therapeutics and several clinical trials are currently trying miRNA molecules [219]. However, specific delivery of these miRNA-based therapies is challenging, if not impossible. The only therapy of this kind investigated today for RCC was MRX34. MRX34 miRNA mimics the tumor suppressor miRNA34 and was tested in a phase I clinical trial for advanced or metastatic RCC and other cancers. Unfortunately, the trial was abandoned early because of serious immunologic adverse events (NCT01829971).

Oblimersen (G3139) is a phosphorothioate antisense oligonucleotide used for chronic lymphocytic leukemia and for advanced melanoma. It targets the sequence around translation initiation of the bcl-2 mRNA inhibiting its translation, resulting in decreased levels of the bcl-2 protein, an apoptotic inhibitor expressed in some types of cancer and linked to tumor drug resistance. Therefore, this target has the potential to enhance the efficacy of standard cytotoxic chemotherapy. In RCC cells, oblimersen induced a specific downregulation of Bcl-2, mainly through a Fas-dependent pathway, and was considered a potential therapy for metastatic RCC in combination with IFN-α [220]. However, a phase II study with oblimersen and IFN-α in metastatic RCC revealed oblimersen did not appear sufficiently active to warrant further development in advanced RCC [193].

GTI-2040 is another antisense agent that targets the small subunit component of human ribonucleotide reductase and displays potent anti-tumor activity against different neoplasia [221]. A synergistic effect with IFN-α for apoptosis and decreased proliferation was suggested [192]. However, a phase I/II study of GTI-2040 and capecitabine in patients with RCC gave very disappointing results [222].

Tazemetostat (EPZ-6438), a EZH2 selective inhibitor, was approved for the treatment of advanced epithelioid sarcoma and its effect in enhancing the therapeutic response to 5-fluorouracil in colorectal cancers has been recently confirmed [223]. Other EZH2 inhibitors are now under clinical evaluation and offer alternative approaches to target this HMT [224]. lncRNAs are also a promising source to develop new target therapies in the future. Many deregulated lncRNA interact with EZH2 to silence TSGs and to induce EMT. As a result, inhibitors of EZH2 and consequently H3K27 methylation remain a very interesting opportunity to develop future RCC therapies [149].

Another opportunity of epigenetic therapy stands in the phenomenon of synthetic lethality that describes a relationship between two genes, the loss of which is incompatible with cell survival. So, contrary to gain-of-function mutations in oncogenes, loss-of-function mutations in TSGs are even more challenging to approach from the therapeutical perspective. Loss-of function mutations in chromatin modifiers has several theoretical applications. For example, loss of *SETD2* becomes synthetically lethal with loss of mitotic inhibitor protein kinase Wee1 [113], loss of *BAP1* is synthetically lethal with simultaneous inhibition of EZH2 or PRC2 [225], and a third mechanism is loss of *PBRM1*, ARID1A, and some components of the SWI/SNF complex, together with inhibition of EZH2 [44,226]. Additionally, *PBRM1* loss promotes immunogenicity in RCC by activation of IFN-responsive genes and probably also confers sensitivity to immune checkpoint inhibitors [44]. Hopefully future developments can take advantage of the improved knowledge in epigenetic modifiers activity in ccRCC to support new therapeutic approaches.

### 3.4. Caveats and Limitations of Epigenetic Therapy

Targeting the epigenome appears an attractive treatment option for RCC because the epigenetic dysregulation of this neoplasia is very extensive and affects many different signaling pathways and tumor hallmarks. The classical concept of an epigenetic therapy centers on the restoration of a neoplastic epigenetic pattern to a normal one. However, the initial therapeutic experience with the drugs available today has been certainly disappointing.

Epigenetic therapy has a robust preclinical base, but many problems remain and need be solved before its generalization. The most important limitation is the lack of selectivity because epigenetic events are ubiquitously distributed across normal and cancer cells. Cancer cells can be sensitive to this regulation, but normal cells have the ability to compensate for these epigenetic changes [227]. Besides, demethylating agents not only restore the expression of genes that have been aberrantly silenced during tumor progression, but also activate genes that are normally repressed by promoter DNA methylation. Another limitation is the need to determine the most important epigenetic alterations for a particular neoplasia. In fact, results of epigenetic therapy in hematologic malignancies are impressive, but not in solid tumors. In addition, all the clinical trials performed are early clinical phase studies, and the number of patients treated with epigenetic therapies and the length of these treatments has been very limited, making difficult the evaluation of long-term safety and real-practice clinical efficacy.

The issue that ccRCC is subject to extensive intra-tumoral heterogeneity is an evident drawback for the development of diagnostic and therapeutic strategies and remains a challenge in modern oncology [10,228]. Multi-regional sequencing has confirmed that renal tumors often harbor different sub-clones that can differ in their spectra of mutations in different epigenetic regulatory tumor suppressor genes. These findings suggest that new therapeutic strategies targeting gene dosage and epigenetic modification should be considered for improved personalized cancer medicine [229]. Single-cell technology and multisite tumor sampling could represent an opportunity to overcome this obstacle [230,231].

The modern paradigm of treatment for metastatic RCC is based on antiangiogenic therapy and combined immune modulation. A realistic potential application of epigenetic therapy today would be to reverse the resistance to treatment with antiangiogenic drugs once they became unresponsive [232,233]. Another promising possibility in treating advanced ccRCC would be the combination of epigenetic drugs and modern immunotherapy using antibodies that block programmed cell death protein 1 (PD1) and its ligands [234]. It would be desirable that epigenetics-based treatments could re-sensitize the host immune response to immunotherapies and restore immunogenicity enforcing the expression of tumor associated antigens, checkpoint ligands in tumor cells, and antigen-processing machinery components [235]. Recent data show that *PBRM1* loss is associated with a less immunogenic tumor microenvironment and upregulated angiogenesis [236]. PBRM1 deficient RenCa subcutaneous tumors in mice are more resistant to ICI, and a retrospective analysis of the IMmotion150 trial also suggests that *PBRM1* mutation reduces benefit from immune checkpoint blockade [151,215].

Nevertheless, the role of PBRM1 mutations in ccRCC in relation to the immune microenvironment is not totally clear. PBRM1 loss of function may alter global tumor-cell expression profiles and influence responsiveness to ICI. Recent studies show truncating mutations in PBRM1 increase the clinical benefit of ICI therapy in patients with metastatic ccRCC [237,238]. PBRM1 alterations have also been clinically validated as marker of ICI responsiveness in RCC but the effect on response and survival is modest and has been mainly observed in the subset of patients who received prior antiangiogenic therapy [239]. The value of PBRM1 mutations in the first-line ICI setting needs further investigation.

The position and results achieved by standard therapies in metastatic ccRCC based on TKIs, m-TORIs, and ICIs, alone or in combination, cannot be easily achieved by other novel therapies. So, epigenetic treatments, via several signaling mechanisms involving both tumor cells and host immune cells, might enhance the efficacy of immune checkpoint therapy in RCC [240]. The combination of epigenetic therapy and immunotherapy is being intensively investigated, and novel trials will be needed to elucidate this role as adjunctive therapy. Epigenetic inhibitors are able to reverse or overcome immune resistance to immunotherapy treatment through upregulation of chemokine expression, antigen processing and presentation machinery, and immune checkpoint molecules [241]. As such, the rationale is that the epigenetic modifiers can be used to prime and sensitize T cells to immunotherapy. Administering “epitherapy” in conjunction with ICI could decrease T-cell exhaustion and avoid immunotherapy resistance.

Additionally, genetic alterations in histone modifier genes in RCC could not only be responsible for the pathogenesis of the disease but also represent potential biomarkers of response to immunotherapies [242]. In this sense, despite the initial failure of epigenetic treatments to reach the clinic, epigenetic therapy is currently a promising strategy for anticancer treatment and for development of new ccRCC tumor markers. However, optimized modern epigenetic treatment options, possibly in combination with other treatments, still remain to be discovered.

## 4. Conclusions

Epigenetic studies have provided a large body of evidence regarding hypermethylated genes, histone-modifying enzymes or miRNAs and new challenges at bench side of patients with RCC. Less invasive diagnosis, histologic subtyping, clinical monitoring of the disease and prognostic evaluation will surely benefit from this increased epigenetic knowledge. However, despite the evidence accumulated, no pure epigenetic biomarker has completed evaluation in phase III studies or is commercially available for clinical use in ccRCC. Prospective multicenter validation is needed before a novel generation of biomarkers become accessible and have the potential to make great strides in personalized medicine. Additionally, early clinical trials have been conducted to evaluate epigenetic therapies for RCC, either alone or in combination with other therapies including IFN-α2b, IL-2, anti-VEGF, TKIs, and mTOR inhibitors. Newer clinical trials are ongoing to investigate the combination of epigenetic treatments with the ICIs pembrolizumab and atezolizumab. There is no doubt that the study of renal cancer epigenetics is still in a formative stage and its application to develop new therapeutic strategies is no more than promising.

## Figures and Tables

**Table 1:** Epigenetic treatments alone or in combination with other treatments used in clinical trials conducted on patients with metastatic or unresectable renal cell carcinoma, or in advanced solid tumors including renal cell carcinoma (clinicaltrials.gov, accessed on 1 March 2021). HDAC: Histone deacetylase; DNMT: DNA methyltransferase. Ref.: reference number as cited in the text.

Epigenetic Drug	Combined Therapy	Phase	Trial Registry	Ref.
**HDAC Inhibition**				
Vorinostat	-	II	NCT00278395	-
Vorinostat	Isotretinoin	I/II	NCT00324740	-
Vorinostat	Bevacizumab	I/II	NCT00324870	[177]
Vorinostat	Sirolimus	I	NCT01087554	[178]
Vorinostat	Ridaforolimus	I	-	[179]
Vorinostat	Pembrolizumab	I	NCT02619253	-
Panobinostat	Sorafenib	I	NCT01005797	-
Panobinostat	-	II	NCT00550277	[180]
Panobinostat	Everolimus	I/II	NCT01582009	[181]
Entinostat	Isotretinoin	I	-	[182]
Entinostat	IL-2	I/II	NCT01038778	[183]
Entinostat	IL-2	I/II	NCT03501381	-
Entinostat	Atezolizumab plus Bevacizumab	I/II	NCT03024437	-
Entinostat	Nivolumab plus Ipilimumab	II	NCT03552380	-
Depsipeptide	-	II	-	[184]
Romidepsin	-	I	NCT01638533	-
Romidepsin	-	II	NCT00106613	[185]
Belinostat	-	I	NCT00413075	[186]
**DNMT Inhibition**				
Azacytidine	IFN-α	I	NCT00217542	-
Azacytidine	Valproic Acid	I	-	[187]
Azacytidine	Bevacizumab	I/II	NCT00934440	-
Decitabine	-	I	-	[188]
Decitabine	IL-2	I	-	[189]
Decitabine	IFN-α	II	NCT00561912	-
Decitabine	Anti-PD-1	I/II	NCT02961101	-
Decitabine	MBG453	I	NCT02608268	-
Decitabine	Oxaliplatin	II	NCT04049344	-
Oligonucleotide MG98	-	I/II	NCT00003890	[190]
Oligonucleotide MG98	IFN-α	I/II	-	[191]
**Other Therapeutic Strategies**				
miRNA MRX34	-	I	NCT01829971	-
Oligonucleotide GTI-2040	Capecitabine	I/II	NCT00056173	[192]
Oligonucleotide Oblimersen	IFN-α	II	NCT00059813	[193]

## Data Availability

Full data will be available from the Corresponding Author upon reasonable request.
